# Clinicopathological differences in focal segmental glomerulosclerosis depending on the accompanying pathophysiological conditions in renal allografts

**DOI:** 10.1007/s00428-023-03703-6

**Published:** 2023-11-18

**Authors:** Sekiko Taneda, Kazuho Honda, Junki Koike, Naoko Ito, Hideki Ishida, Toshio Takagi, Yoji Nagashima

**Affiliations:** 1 Department of Surgical Pathology, Tokyo Women’s Medical University, 8-1, Kawada-Cho, Shinjuku-Ku, Tokyo, 162-8666 Japan; 2 Department of Anatomy, Showa University School of Medicine, Tokyo, Japan; 3 Department of Pathology, St. Marianna University School of Medicine, Kawasaki, Kanagawa Japan; 4 Department of Organ Transplant Medicine, Tokyo Women’s Medical University, Tokyo, Japan; 5 Department of Urology, Tokyo Women’s Medical University, Tokyo, Japan

**Keywords:** Renal transplant biopsy, Focal segmental glomerulosclerosis, Recurrence, Antibody-mediated rejection, Calcineurin-inhibitor, Colombia classification

## Abstract

Primary focal segmental glomerulosclerosis (FSGS) is thought to be caused by circulating factors leading to podocytopathy, whereas segmental sclerotic lesions (FSGS lesions) have several causes. We studied the clinicopathological differences of FSGS-lesions in 258 cases of FSGS in renal allografts, depending on the following accompanying pathophysiology: recurrence of primary FSGS, calcineurin inhibitor (CNI)-induced arteriolopathy, antibody-mediated rejection (ABMR), and other conditions. All cases were categorized with the Columbia classification. Recurrent FSGS developed the earliest after transplantation and showed the highest percentage of the collapsing (COL) variant in which collapse of the glomerular capillaries with epithelial hypertrophy was apparent. FSGS accompanying CNI-induced arteriolopathy predominantly developed the not otherwise specified (NOS) variant, showing severe ultrastructural endothelial injury. On the contrary, approximately 7% of the cases showed the COL variant, presenting glomerular endothelial damage such as double contours of glomerular basement membrane and endothelial cell swelling as well as epithelial cell proliferation. FSGS with ABMR had the highest creatinine levels and cellular variant percentage, with marked inflammation and ultrastructural endothelial injury. Approximately two-thirds of the cases without ABMR, CNI-induced arteriopathy, or recurrent FSGS had other coexisting conditions such as glomerulonephritis, T cell-mediated rejection, and reflux nephropathy with progressive tubulointerstitial fibrosis. Most of these cases were of the NOS variant. The clinicopathologic features of post-transplant FSGS differed depending on the associated conditions, and endothelial injury was apparent especially in cases of CNI-induced arteriolopathy and ABMR. Precise observation of FSGS lesions may facilitate the diagnosis and clinical management of FSGS during renal transplantation.

**Supplementary Information:**

The online version contains supplementary material available at 10.1007/s00428-023-03703-6.

## Introduction

Focal segmental glomerulosclerosis (FSGS) is a morphological glomerular injury category. Primary FSGS is considered to be podocytopathy caused by circulating factors such as urokinase receptor and cardiotropin-like cytokine-1 [1, 2]. Primary FSGS often manifests as steroid-resistant nephrotic syndrome and frequently recurs after transplantation [3]. In contrast, secondary FSGS has several causes with diverse clinical manifestations. FSGS lesions are also known to result from renal disorders such as glomerulonephritis, arterionephrosclerosis or any injury that substantially decreases nephron numbers [4]. Furthermore, FSGS lesions may manifest secondary to severe advanced primary tubulointerstitial diseases, such as chronic urinary tract obstruction or pyelonephritis [4].

Renal allografts have clear differences from native kidneys in the use of immunosuppressive drugs and rejection. Calcineurin inhibitors (CNI) are effective immunosuppressive agents for renal transplantation, targeting glomerular and peritubular capillary endothelial cells [5]. However, long-term CNI use can induce arteriolopathy in which hyaline material deposits replace the degenerated vascular smooth muscle [6], causing local ischemia. Antibody-mediated rejection (ABMR) is caused by anti-donor human leukocyte antigen specific antibodies (DSA) and is histologically defined by microvascular inflammation in peritubular and glomerular capillaries. Double contours of the glomerular basement membrane (GBM) indicate a chronic change in ABMR, leading to graft dysfunction.

Although primary FSGS is primarily due to podocyte injury, endothelial cell injury may also contribute to the segmental glomerular sclerosis in human FSGS cases [7, 8], as shown in an experimental model of collapsing FSGS [9], where podocyte injury caused endothelial damage by local crosstalk signaling [9]. In this study, we classified 258 cases of FSGS in renal allografts according to the cause of the renal failure and the accompanying pathological diseases seen on biopsy, including CNI-induced arteriolopathy, and ABMR. We also evaluated the clinicopathological differences of the segmental lesions.

## Materials and methods

### Biopsy sample selection

This study involved the analysis of 3,762 renal allograft biopsies conducted at Tokyo Women’s Medical University between April 2008 and March 2016. Of these, 299 cases (7.9%) exhibited segmental lesions in the glomeruli. The cases with segmental lesions were divided into four groups: those with FSGS as the original renal disease causing renal failure (recurrent-FSGS group), those with moderate-to-severe CNI-induced arteriolopathy (Banff aah score ≥ 2) [6] in biopsy specimens (CNI-FSGS group), those with ABMR in biopsy specimens (ABMR-FSGS group), and those not belonging to any of these groups (unknown etiology [UE]-FSGS group). After excluding 40 cases whose characteristic overlapped between two or more groups, 258 cases were included in this study. The original diseases that caused renal failure remained unknown in 62 cases (six cases in ABMR-FSGS, 22 in CNI-FSGS, and 34 in UE-FSGS groups). The patients’ data were obtained from medical records, and informed consent was obtained using the opt-out method. The study was conducted in compliance with the guidelines stipulated in the Declaration of Helsinki and was approved by the ethics committee at Tokyo Women’s Medical University (No. 5407).

### Histologic evaluation

All biopsies were stained with hematoxylin and eosin, periodic acid-Schiff (PAS), Masson’s trichrome, and periodic acid-methenamine silver (PAM) for standard light microscopy analysis. Immunofluorescence was performed using 2-μm cryostat sections and fluorescein isothiocyanate-conjugated polyclonal antibodies against IgG, IgA, IgM, C3c, C1q, C4d and κ and λ light chains. The C4d staining of the peritubular capillary and Banff lesion scores were evaluated based on the Banff 2017 classification [10], and pathological diagnosis was independently confirmed by two pathologists. The percentages of tubulointerstitial fibrosis and tubular atrophy (IF/TA) in the cortex were visually determined following the Banff classification. Arteriosclerosis was categorized based on the extent of involvement of the internal elastic membrane involvement: 0, none; 1, mild; 2, moderate; and 3, severe. Reflux nephropathy was diagnosed based on clinical information or histopathological findings such as medullary ray injury with thyroidization and/or Tamm-Horsfall protein deposition in the glomerulus. Additionally, cases were classified into five histological variants according to Columbia classification [11]: collapsing (COL), tip (TIP), cellular (CEL), perihilar (PH), and not otherwise specified (NOS). The COL, TIP, and CEL variants predominantly exhibited histological activity, whereas the PH and NOS variants showed histological chronicity [11]. This classification corresponds well with the clinical features and prognosis of FSGS [12, 13].

Specimens for electron microscopy (EM) were obtained at the time of biopsy. EM was performed at the discretion of the pathologists who made the initial diagnosis, for total 112 of the 258 cases (30 cases in recurrent-FSGS, 19 in ABMR-FSGS, 40 in CNI-FSGS, and 23 in UE-FSGS groups). In the recurrent-FSGS group, EM was performed early, within approximately 2 years after transplantation in 18 cases and 2 or more years post-transplantation in 12 cases. In the CNI-FSGS group, EM was performed, especially in cases without NOS variant was present. In the ABMR-FSGS group, EM was performed in 14 cases with chronic active ABMR and 5 cases with active ABMR. Finally, in the UE-FSGS group, EM was performed especially in the cases without NOS variant or in cases with glomerulonephritis requiring the confirming of electron dense deposits.

### Statistical analysis

Data including post-transplant duration (PTD) to biopsy, follow-up duration, and years from biopsy to hemodialysis (HD) were presented as absolute numbers and median value. Other continuous variables are presented as means ± standard deviation. Between-group differences were analyzed using Student’s *t-*test or the Mann–Whitney *U*-test, and multigroup analyses were performed using one-way analysis of variance. To compare continuous variables between the two groups, multiple comparisons (post hoc) using the Scheffe’s test were performed. JMP Pro software (SAS Institute Japan) was used for all analyses, and *p*-values < 0.05 were considered to indicate statistical significance.

## Results

### Clinical characteristics of each FSGS group

Table 1 presents the clinical characteristics of each group. The recurrent-FSGS group (*n* = 45) had the youngest recipient, and the highest percentage of patients aged < 30 years (37.8%; *p* < 0.01). This group also showed the shortest PTD to biopsy and the highest percentage of patients with nephrotic syndrome (35.6%; *p* < 0.01). Approximately 90% of the cases were detected by episode biopsy. The percentage of cadaveric kidney transplants tended to be higher in the recurrent-FSGS group. The CNI-FSGS group (*n* = 90) had the longest PTD to biopsy (*p* < 0.01), and 72.2% of cases were detected by episode biopsy. In the ABMR-FSGS group (*n* = 28), seven cases were diagnosed with active ABMR and 21 with chronic active ABMR. The ABMR-FSGS group showed the highest percentage of females whose husbands were donors and who had a history of pregnancies with their husbands (31.6%) and the highest serum creatinine (sCr) levels at the time of biopsy; 81.5% of cases were detected by episode biopsy. The UE-FSGS group (*n* = 95) demonstrated the lowest sCr level, and 47.4% of the cases were detected by protocol biopsy. No difference was found in the donor age or percentage of ABO incompatibility cases (data not shown). Overall, 73% of cases were followed up and 37% of the cases were restarted on hemodialysis (HD).

**Table 1:** Clinical characteristics of each FSGS group

	1. Recurrent- FSGS	2. CNI-FSGS	3. ABMR-FSGS	4. UE-FSGS	*p*
N (%)	45 (17.4%)	90 (34.9%)	28 (10.9%)	95 (36.8%)	< 0.001, 1 vs. 2,3,4
Age (years)	32.6 ± 10.8	45.6 ± 10.1	49.2 ± 13.4	46.3 ± 13.6	< 0.01, 1 vs. 2,3,4
Age ≤ 30 (%)	17 (37.8%)	1 (1.1%)	1 (3.6%)	9 (9.5%)	< 0.05, 2 vs. 3,4
Sex (female, %)	23 (51.1%)	30 (32.6%)	19 (70.4%)	26 (27.3%)	*p* < 0.001, 3 vs. 1,2
Husband-to-wife transplantation with a history of pregnancies (%)					
	0 (0%)	0 (0%)	6 (31.6%)	2 (7.7%)	(*p* = 0.0546, 3 vs. 4)
Cadaveric	10 (22.2%)	7 (7.8%)	1 (3.6%)	7 (7.4%)	< 0.05, 1 vs. 2,3,4
PTD to biopsy (years)	1.4 (0.05–11.5)	10.2 (0.3–24.0)	2.7 (0.1–16.1)	2.7 (0.1–18.3)	< 0.01, 2 vs. 1,3,4
Nephrotic syndrome (%)	16 (35.6%)	2 (2.2%)	2 (7.1%)	3 (3.2%)	< 0.01, 1 vs. 2,3,4
sCr (mg/dL)	2.02 ± 1.68	2.04 ± 0.91	2.42 ± 1.48	1.75 ± 0.93	< 0.05, 3 vs. 4
UP (scale 0–4)	2.59 ± 1.37	1.51 ± 1.04	1.57 ± 1.31	1.03 ± 1.11	< 0.01, 1 vs. 2,3,4
Reason for renal biopsy					
Non-episode (Protocol)	5 (11.1%)	25 (27.8%)	5 (18.5%)	45 (47.4%)	< 0.001, 4 vs. 1,2,3*
Increase in proteinuria	20 (44.4%)	15 (16.7%)	1 (3.7%)	10 (10.5%)	< 0.001, 1 vs. 2,3,4*
Increase in serum Cr level	8 (17.8%)	39 (43.3%)	15 (55.6%)	30 (31.5%)	< 0.05, 3 vs. 1,4*
Increase in both proteinuria and Cr level	12 (26.7%)	11 (12.2%)	7 (25.9%)	10 (10.5%)	< 0.05, 1 vs. 4*
F/U case number (n, %)	33 (73.3%)	62 (68.8%),	16 (57.1%)	78 (82.1%)	
F/U years	6.2 (2.7–8.9)	7.0 (0.5–14.9)	5.9 (0.4–11.8)	7.1 (0.1–18.6)	-
HD cases (n, %)	15 (45.4%)	25 (40.3%)	9 (56.3%)	21 (22.1%)	< 0.05, 3 vs. 4
Years from biopsy to HD	3.9 (1.1–8.2)	4.1 (0.9–11.0)	2.9 (0.8–11.8)	4.1 (0.1–18.6)	-

Post-transplant duration (PTD) to biopsy, follow-up (F/U) years, and years from biopsy to hemodialysis (HD), data are presented as absolute numbers and median values. Other data are presented as means ± standard deviations. Abbreviations: FSGS, focal segmental glomerulosclerosis; ABMR, antibody-mediated rejection; CNI, calcineurin-inhibitor; UE, unknown etiology; sCr, serum creatinine; UP, urinary proteinuria. *Tests were performed using the ratio of protocol biopsies (or increase in proteinuria and/or serum Cr levels) to episodic biopsies (or no increase in proteinuria and/or serum Cr levels)

### Pathological characteristics of each FSGS group

**Fig. 1:**
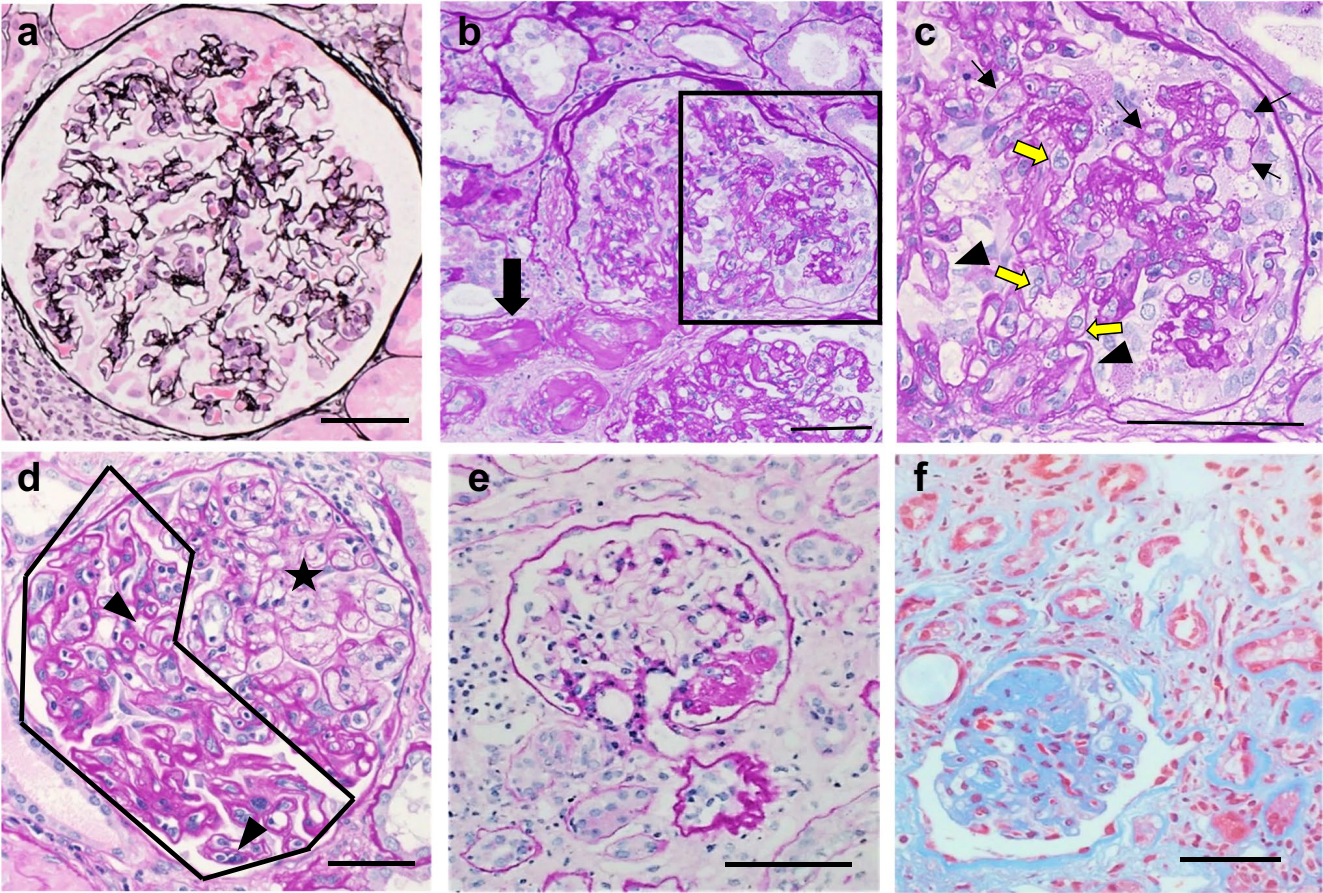
Light micrographs of glomeruli. **a** Recurrent-FSGS group; **b** and **c** CNI-FSGS group; **d** ABMR-FSGS group; e and f UE-FSGS group. **a** Recurrent-FSGS group, COL variant (UP, 2 + ; serum Cr, 1.81 mg/dL; 75 days after transplantation): epithelial hypertrophy and bridging epithelial cells from the tuft to the Bowman’s capsule with capillary loop collapse. Some epithelial cells show an abundant cytoplasmic droplet. Approximately 6 years after the biopsy, hemodialysis (HD) was initiated. (PAM staining, scale bar = 50 μm). **b** and **c** CNI-FSGS group, COL variant (UP, 2 + ; serum Cr, 2.61 mg/dL; 13 years after transplantation): **b** Narrowed afferent arterioles with severe hyaline deposition due to CNI arteriolopathy (arrow). Glomeruli show segmental capillary collapse with epithelial cell proliferation (black square). **c** shows the black square in **b**. Double contours of GBM (arrow heads), endothelial cell swelling (yellow arrows), and intracapillary foam cells (black arrows), suggestive of endothelial cell damage. Approximately 6 years after the biopsy, HD was initiated. **b**, **c**: PAS staining, scale bars = 50 μm). **d** ABMR-FSGS group, CEL variant (UP, 2 + ; serum Cr, 1.61 mg/dL; 2.7 years after transplantation): segmental expansion of the glomerular capillary by many foam cells, occluding the capillary lumina (star), is noted. Severe glomerulitis and double contours of GBM (arrow heads), suggestive of chronic active ABMR (area surrounded by black line). Approximately 6 months after the biopsy, the patient’s renal function was stable (serum Cr level was 1.51 mg/dL without proteinuria). (PAS staining, scale bar = 50 μm). **e** UE-FSGS group, PH variant (UP, ± ; serum Cr, 1.79 mg/dL; 9 months after transplantation): this case was diagnosed with acute T cell-mediated rejection at 3 months after transplantation, and follow-up biopsy was performed 6 months later. The vascular rejection, which was not seen in the previous biopsy, is observed in this follow-up biopsy (cv1, v1), suggesting that T cell-mediated rejection has persisted. The glomerulus shows segmental perihilar sclerosis. The IF/TA is 30% of the cortex. Arteriosclerosis is moderate, and arteriolar hyalinosis is absent. Approximately 5 years after the biopsy, the patient’s renal function was stable (serum Cr, 1.54 mg/dL without proteinuria). (PAS staining, scale bar = 100 μm). **f** UE-FSGS group, NOS variant (serum Cr, 2.12 mg/dL without proteinuria, 0.5 years after transplantation): reflux nephropathy was diagnosed clinically and confirmed pathologically with findings of medullary ray injury and fibrosis with glomerular deposition of Tamm–Horsfall protein. There is segmental expansion of the mesangial matrix with capillary collapse. The IF/TA is 20% of the cortex. There is no arteriosclerosis or arteriolar hyalinosis. Approximately 10 years after the biopsy, the patient’s renal function was stable (serum Cr 1.69 mg/dL without proteinuria). (Masson’s trichrome staining, scale bar = 50 μm)

**Fig. 2:**
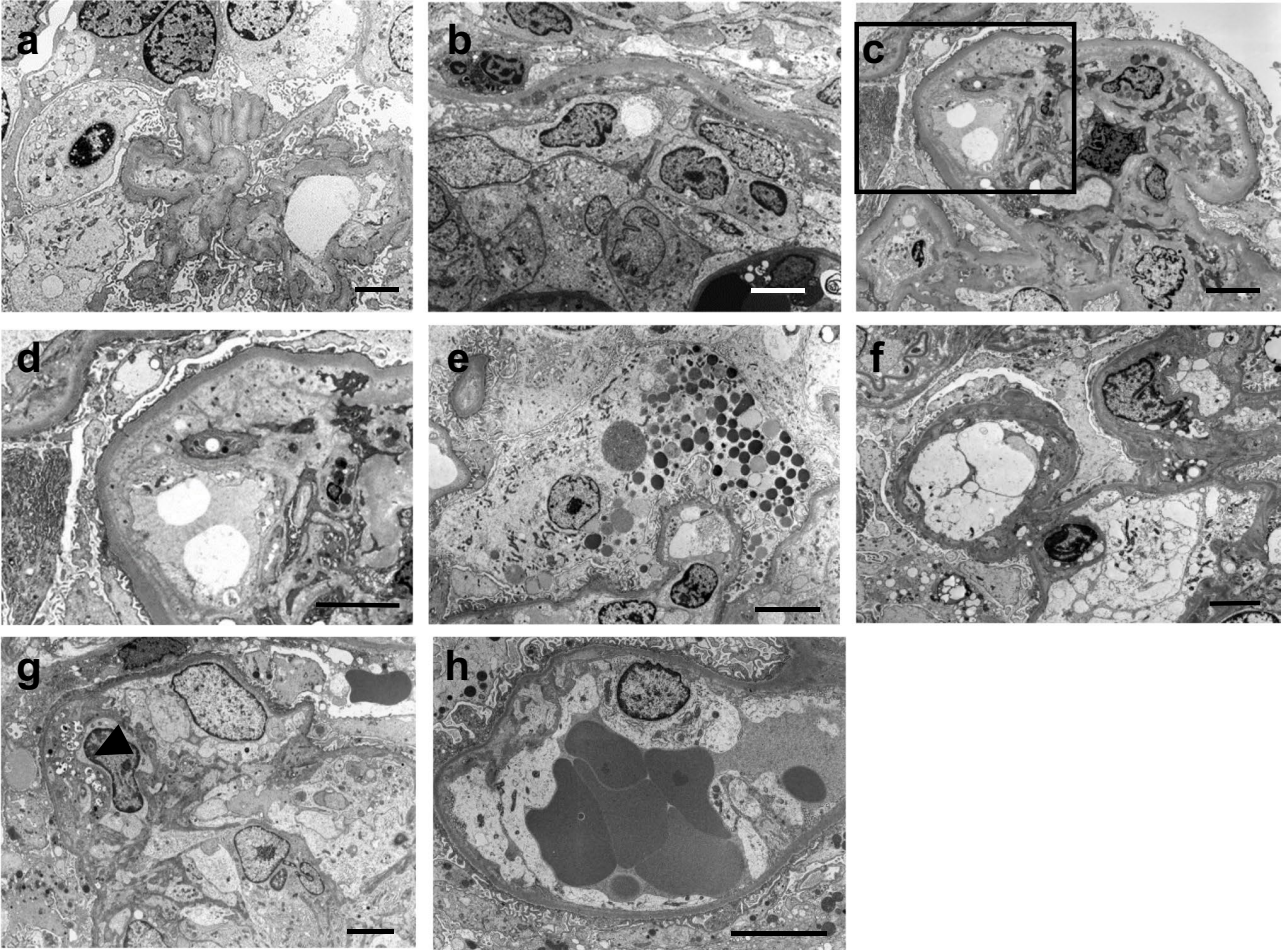
Electron micrographs of glomeruli. **a** and **b** Recurrent-FSGS group; c- f, CNI-FSGS group; **g** and **h** ABMR-FSGS group. **a** and **b**: Recurrent-FSGS group, COL variant (UP 4 + with serum Cr 1.00 mg/dL; 55 days after transplantation): **a** The epithelial cells around the collapsed capillaries are hypertrophied. Endothelial damage, such as subendothelial widening, is focally observed. **b** The proliferating cells fill Bowman’s space. Neither foot process nor aggregation of actin filaments was detected at the base of the epithelial cells. Six years after the biopsy, the patient’s renal function was deteriorating (serum Cr was 1.40 mg/dL with UP3 +). (**a**, **b**: scale bars = 5 μm). **c** and **d**: CNI-FSGS group, NOS variant (UP + with an increase of serum Cr 1.60 mg/dL; 10 years after transplantation): **c** The subendothelial space is enlarged (black square), and exudative changes with mesangial expansion are prominent. **d** shows the black square in (**c**). Mild podocyte injury, including focal detachment and degeneration is seen. Approximately 3 years after the biopsy, HD was initiated. (**c**, **d**: scale bars = 5 μm). **e** and **f**: CNI-FSGS group, COL variant (UP + with an increase of serum Cr 1.55 mg/dL; 1 year after transplantation): **e** The epithelial cells around the collapsed capillaries are hypertrophied, and protein droplets are sometimes seen in the cytoplasm. **f** the same case as in (**e**). The foot process effacement is partially apparent, and there is marked endothelial swelling. Approximately 4 years after the biopsy, the patient’s renal function remained stable (serum Cr was 1.60 mg/dL with UP +). (e, f: scale bars = 5 μm). **g** and **h**: ABMR-FSGS group, CEL variant (serum Cr 1.21 mg/dL without proteinuria; 1-year protocol biopsy): **g** Many inflammatory and foam cells (arrowhead) occlude the capillary. The foot process is focally effaced. **h** the same case as in **g**. Endothelial injury, visible as endothelial swelling and subendothelial widening, is prominent. Approximately 10 months after the biopsy, the patient’s renal function was stable (serum Cr was 1.30 mg/dL without proteinuria). (**g**, **h**: scale bars = 5 μm). FSGS, focal segmental glomerulosclerosis; CNI, calcineurin inhibitor; ABMR, antibody-mediated rejection; UE, unknown etiology; COL, collapsing; CEL, cellular; PH, perihilar; and NOS, not otherwise specified

Table 2 presents the pathological characteristics of each group. The typical light and electron microscope images of each variant are shown in Fig. 1. The recurrent-FSGS group demonstrated the lowest rate of global sclerosis (18.6 ± 17.2%) and interstitial fibrosis and tubular atrophy (IF/TA) (16.7 ± 12.9%) among the groups. The percentage of segmental sclerotic glomeruli did not significantly differ among the groups. The COL variant incidence was the highest among all groups (46.7%). Supplemental Fig. 1 illustrates the relationship between PTD, histological variant, and prognosis restricted to cases with severe proteinuria (urinary protein [UP] ≥ 3). The percentage of COL variant was high up to approximately 70% (Supplemental Fig. 1). In particular, when severe proteinuria occurred within 3 months after transplantation, all cases showed the COL variant, whereas the NOS variant became more common 2 years after transplantation (Supplemental Fig. 1). HD reintroduction was also the highest in COL variant cases. Microscopy revealed epithelial cell proliferation which sometimes formed bridges between the Bowman’s capsule and glomerular tuft with capillary loop collapse in COL variant cases (Fig. 1a). On electron micrographs, the collapse of the glomerular capillary with epithelial hyperplasia and hypertrophy was apparent (Fig. 2a). The proliferating cells that filled Bowman’s space showed no foot processes or actin filament rearrangement (Fig. 2b). Mild endothelial injury, such as subendothelial widening, was observed mainly in the area of collapsed capillaries (Fig. 2a).

**Table 2:** The pathological characteristics of each FSGS group

	1. Recurrent- FSGS	2. CNI-FSGS	3. ABMR-FSGS	4. UE-FSGS	p
N (%)	45 (17.4%)	90 (34.9%)	28 (10.9%)	95 (36.8%)	
Global sclerosis (%)	18.6 ± 17.2	33.6 ± 20.9	22.0 ± 15.1	21.8 ± 16.0	< 0.001, 2 vs. 1,3,4
Segmental sclerosis (%)	11.7 ± 11.1	10.4 ± 10.2	12.3 ± 10.0	10.3 ± 10.6	-
FSGS variant COL	21 (46.7%)	6 (6.7%)	4 (14.2%)	5 (5.3%)	< 0.05, 1 vs. 2,3,4*
TIP	1 (2.2%)	1 (1.1%)	2 (7.1%)	3 (3.1%)	-*
CEL	6 (13.2%)	6 (6.7%)	9 (32.1%)	12 (12.6%)	< 0.05, 3 vs. 2,4*
PH	3 (6.6%)	6 (6.7%)	1 (3.5%)	6 (6.3%)	-*
NOS	14 (31.1%)	71 (78.8%)	12 (42.6%)	69 (72.6%)	< 0.05, 2 or 4 vs. 1,3*
C4d on peritubular capillary (score: 0–3)	0.56 ± 1.02	0.45 ± 0.80	1.00 ± 1.13	0.86 ± 1.00	< 0.001, 3 vs. 2
g (score: 0–3)	0.13 ± 0.42	0.06 ± 0.35	1.54 ± 0.96	0.02 ± 0.15	< 0.0001, 3 vs. 1,2,4
ptc (score: 0–3)	0.27 ± 0.46	0.14 ± 0.05	1.75 ± 0.80	0.24 ± 0.58	< 0.0001, 3 vs. 1,2,4
cg (score: 0–3)	0.02 ± 0.20	0.23 ± 0.56	1.36 ± 1.19	0.07 ± 0.30	< 0.0001, 2 vs. 1,3,4
aah (score: 0–3)	0.40 ± 0.69	2.41 ± 0.50	0.25 ± 0.42	0.29 ± 0.45	< 0.0001, 2 vs. 1,3,4
Arteriosclerosis (score: 0–3)	1.60 ± 0.73	1.86 ± 0.64	1.69 ± 0.71	1.39 ± 0.77	< 0.05, 2 vs. 1,4
IF/TA (%)	16.7 ± 12.9	30.4 ± 16.3	19.8 ± 13.3	25.1 ± 14.2	< 0.01, 2 vs. 1,3,4
Comorbidity at the biopsy					
Glomerulonephritis (%)	2 (4.6%)	29 (24.4%)	3 (10.7%)	37 (38.9%)	< 0.01, 2 or 4 vs. 1
IgAN/MN/DMN/others (n)	0/1/0/1	25/0/2/2	0/3/0/0	31/5/1/0	NA
Reflux nephropathy (%)	0	1 (1.1%)	0	13 (13.7%)	< 0.01, 4 vs. 1,2,3
T cell-mediated rejection (%)	4 (9.2%)	0	0	11 (11.6%)	< 0.01, 4 vs. 1,2,3

Data are presented as means ± standard deviations. Abbreviations: FSGS, focal segmental glomerulosclerosis; ABMR, antibody-mediated rejection; CNI, calcineurin-inhibitor; UE, unknown etiology; g, glomerulitis; ptc, peritubular capillaritis; cg, double contours of glomerular basement membrane; aah, hyaline arteriolar thickening; IF/TA, interstitial fibrosis and tubular atrophy; IgAN, IgA nephropathy; MN, membranous nephropathy; DMN, diabetic nephropathy; COL, collapsing; CEL, cellular, PH, perihilar; NOS, not otherwise specified; NA, not available. Tests were performed using the ratio of COL (or TIP or CEL or PH or NOS) variants to nonCOL (or nonTIP or nonCEL or nonPH or non-NOS) variants

The CNI-FSGS group showed the highest global sclerosis rate (33.6 ± 20.9%, *p* < 0.001) and IF/TA (30.4 ± 16.3%, *p* < 0.01) and the most advanced arteriosclerosis (1.86 ± 0.64). Approximately 80% of the cases showed an NOS variant. The typical ultrastructural findings of the NOS variant included prominent subendothelial widening with exudative changes (Fig. 2c, d). Epithelial cell injury including podocyte detachment and degeneration, was focally observed (Fig. 2d). In contrast, 6.7% of the cases showed a COL variant. Microscopy revealed narrowed afferent arterioles with severe hyaline deposition due to CNI-arteriolopathy, and there was segmental capillary collapse with epithelial cell proliferation in the glomeruli (Fig. 1b). Double GBM contours, endothelial cell swelling, and intracapillary foam cell infiltration suggestive of endothelial cell damage, were also observed (Fig. 1c). Ultrastructurally, in the COL variant, the epithelial cells around the collapsed capillaries were hypertrophied, and protein droplets were sometimes seen in the cytoplasm (Fig. 2e). The foot process effacement was focal, and there was marked endothelial swelling (Fig. 2f).

The ABMR-FSGS group demonstrated the severe glomerulitis and double contoured GBM (1.54 ± 0.96 and 1.36 ± 1.19, respectively; *p* < 0.0001) and peritubular capillaritis. This group also had the highest scores of C4d staining along the peritubular capillaries (1.00 ± 1.13). The incidence of the CEL variant (32.1%) was the highest among the groups. Intracapillary foam cell infiltration suggestive of the CEL variant was observed against a background of severe glomerulitis and double GBM contours, suggestive of chronic active ABMR (Fig. 1d). Severe intracapillary inflammation and foam cell infiltration was seen on electron micrographs (Fig. 2g), with prominent endothelial injury, as reflected by endothelial swelling and subendothelial widening (Fig. 2h). There was focal foot process effacement with podocyte degeneration (Fig. 2g, h).

The UE-FSGS group presented with the second worst degree of IF/TA (25.1% ± 14.2%) following the CNI-FSGS group, whereas the degree of arteriosclerosis was mildest (1.39 ± 0.77). Approximately 80% of the cases showed NOS and perihilar (PH) variants. Overall, 64% of cases had coexisting conditions, such as glomerulonephritis, T cell-mediated rejection (TCMR), or reflux nephropathy. Approximately 40% of cases presented with glomerulonephritis, and IgA nephropathy was the most common form observed at the time of biopsy (84%). Among the 34 cases with unknown primary renal disease in the UE-FSGS group, 6 cases were found to have IgA nephropathy, and two cases had membranous nephropathy, indicating a recurrence rate of 68% for IgA nephropathy. Furthermore, 11.6% and 13.7% of cases exhibited coexisting TCMR (Fig. 1e) and reflux nephropathy (Fig. 1f), respectively, showing NOS or PH variants against a background of advanced interstitial fibrosis.

We determined whether the degree of proteinuria (median and interquartile range) in histological variants was similar to that in native kidneys (Supplemental Fig. 2). In the recurrent-FSGS group, the degree of proteinuria was more pronounced in the COL and CEL variants. The NOS variant exhibited the next highest level of proteinuria, whereas the PH variant exhibited the lowest level of proteinuria (Supplemental Fig. 2a). The observed trend was similar to that reported in FSGS in native kidney [13]. Although a similar trend was also found in the CNI-FSGS group (Supplemental Fig. 2b), the number of with COL and CEL variants was small and the variability was noticeable, hindering the ability to draw general conclusions. Conversely, in the ABMR-FSGS group, where endothelial injury was prominently involved in FSGS-lesions, proteinuria was more common in those with the NOS variant, whereas the degree of proteinuria varied in those with the CEL variant (Supplement Fig. 2c). In the UE-FSGS group, which also harbored various other renal injuries, the degree of proteinuria was limited in all but the COL variant (Supplement Fig. 2d).

**Fig. 3:**
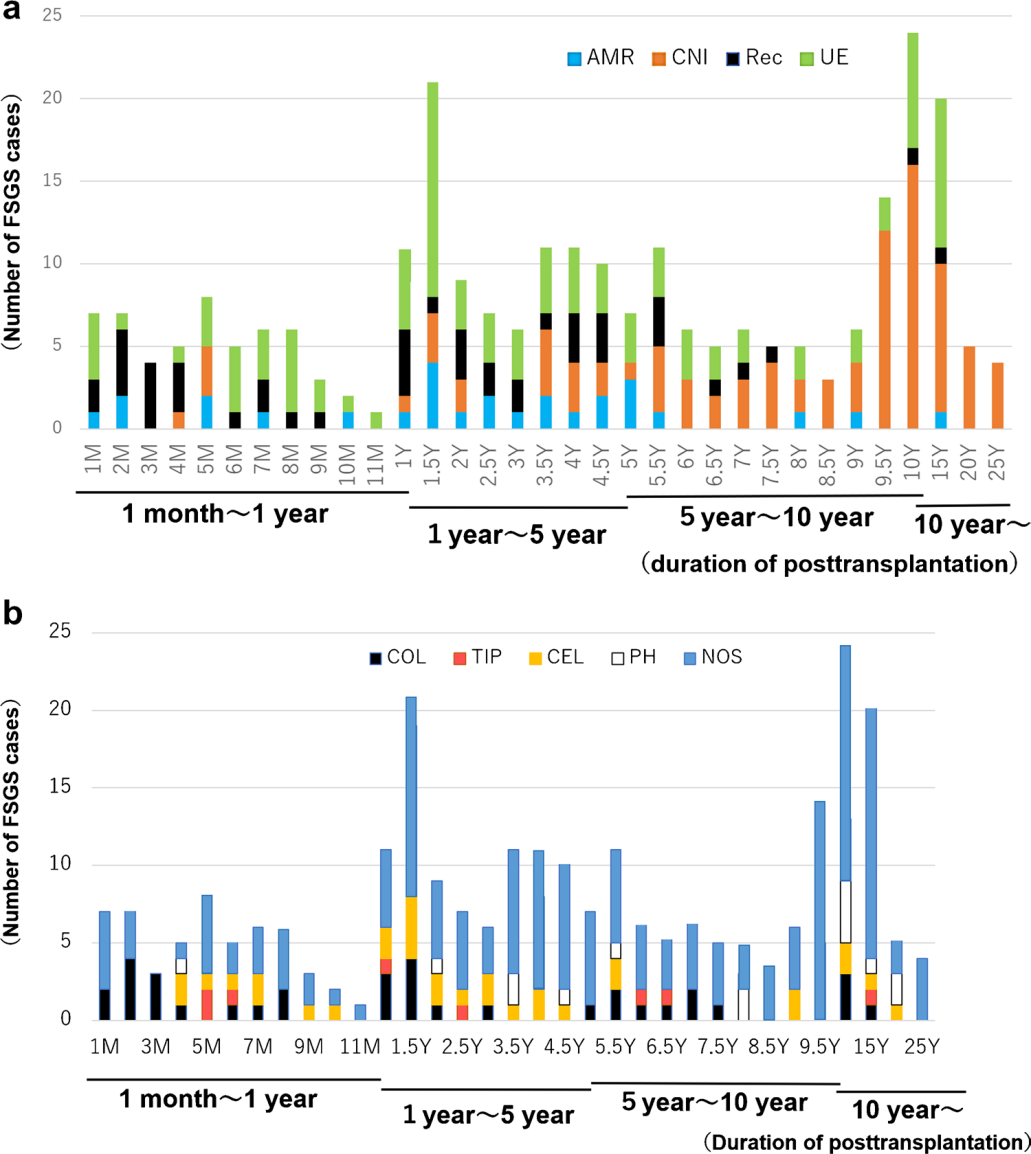
**a** Cases of FSGS categorized by etiology and time after renal transplant. **b** Cases of FSGS categorized by histological variant and time after renal transplant

The FSGS cases categorized by causes and by time after renal transplant are shown in Fig. 3a. ABMR-FSGS and recurrent-FSGS cases were present soon after transplantation and after 1 year, whereas CNI-FSGS cases tended to be more common after 3.5 years, and UE-FSGS cases were present at all-time points (Fig. 3a). The histologic variants were not time-related, making it difficult to identify etiology histologically (Fig. 3b).

## Discussion

**Fig. 4:**
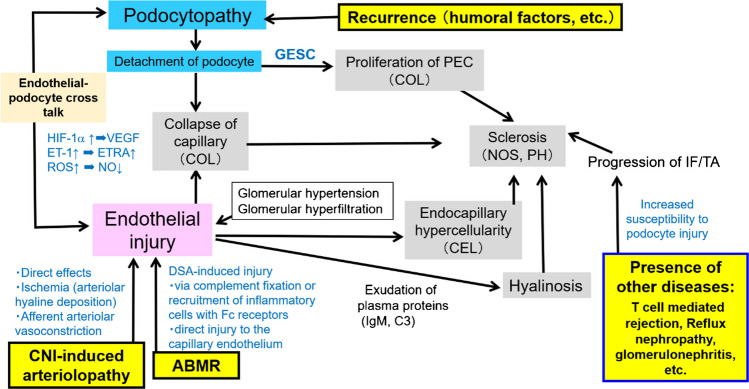
Mechanisms of the development of FSGS lesions in renal allografts. Recurrent FSGS in renal allografts mainly results from podocyte injury caused by humoral factors. The COL variant occurs when activated PECs proliferate and replace podocytes in areas where they have degenerated and detached. The collapsed area may develop segmental sclerosis due to increased matrix. In renal allografts, the hyperfiltration status due to a single kidney may damage the endothelium; CNI-induced arteriolopathy or ABMR may exacerbate this. CNI-FSGS can cause ischemia due to vascular toxicity or direct endothelial cell injury. The endothelial damage caused by ABMR is due to DSA, and the induction of intracapillary inflammation by DSA and plasma protein exudation may lead to CEL and NOS variants. There is crosstalk between endothelial and epithelial cells, which affect each other via cytokines. Furthermore, preexisting interstitial fibrosis may increase podocytes susceptibility to injury and induce FSGS. Thus, both epithelial and endothelial injury contribute to FSGS lesion formation in renal allografts, but the degree of involvement depends on the etiology. FSGS, focal segmental glomerulosclerosis; CNI, calcineurin inhibitor; ABMR, antibody-mediated rejection; COL, collapsing; CEL, cellular; PH, perihilar; NOS, not otherwise specified; GESC, glomerular endothelial stem cell; PEC, parietal epithelial cell; HIF-1α, hypoxia-inducible factor-1α; VEGF, vascular endothelial growth factor; ET-1, endothelin-1; ETRA, endothelin receptor A; ROS, reactive oxygen species; NO, nitric oxide; DSA, donor specific antibody; TCMR, T cell-mediated rejection; IF/TA, interstitial fibrosis and tubular atrophy

The recurrent-FSGS group showed the highest incidence of the COL variant among the groups, which was even higher when limited to cases with severe proteinuria, especially those within 3 months of transplantation. The pathological characteristics of the COL variant were the cell bridges, where the foot processes and rearrangement of actin filaments could not be confirmed. Glomerular epithelial stem cells (GESCs) near the urinary pole of Bowman’s capsule are thought to have the potential to regenerate podocytes, as identified by the surface markers CD24^+^ and CD133^+^ [14, 15]. When podocytes are rapidly lost, GESCs form a cell bridge and promptly replace the lost podocyte (Fig. 4) [14, 16, 17]. The glomerular structure is distorted at the podocyte injury sites, which alters the polarity of GESC division and initiates an abnormal proliferation [14]. These proliferating epithelial cells may be activated parietal epithelial cells (PECs), as they are ectopically positive for PEC markers (Pax-2, Claudin-1, and CK18/8) and CD44 [18]. Epithelial phenotype alterations occur to a much lesser degree in other FSGS variants with podocyte loss [18]. Interestingly, a study using an experimental model reported that activation of PEC prevented leakage of proteins, suggesting that PEC may be protective at the micro level [19]. Furthermore, mild endothelial injury was observed ultrastructurally. Podocytes are the only cells that produce vascular endothelial growth factor (VEGF) in the glomerulus, which is necessary for the survival and function of its endothelial cells. In an vivo study, the loss of podocyte VEGF-A results in thrombotic microangiopathy, whereas its overexpression causes the COL variant of FSGS and endothelial injury [20]. This indicates that the dysregulation of the signaling pathway from podocyte to endothelial cells due to down-regulated VEGF expression might cause the endothelial injury of these lesions (Fig. 4).

The histological variants that observed in native kidneys are similar to that observed in the renal allografts of patients with FSGS. However, the early histologic findings of recurrent FSGS shortly after transplantation are similar to minimal change disease (MCD), i.e., normal light microscopy with moderate/severe foot process effacement on electron microscopy [21]. Subsequently, the detachment of podocytes leads to segmental sclerosis and glomerulosclerosis in patients who are unable to achieve remission [22]. FSGS and MCD are well-known podocytopathies that are also considered to exist along the spectrum of podocyte injury [21]. The present study did not include MCD-like cases. However, similar to previous reports [21, 22], serial biopsy specimens revealed that MCD-like lesions preceded FSGS lesions (Supplemental Fig. 3); this finding may be considered an important clue to the pathogenesis of recurrent FSGS after transplantation.

Mutations in structural podocyte genes cause FSGS with severe nephrotic syndrome at birth or during childhood. Because of their similar clinical presentations, they are often diagnosed as primary FSGS caused by circulating factors, unless genetic testing is performed. No patient in the recurrent-FSGS group had information on genetic abnormalities in their medical records. Genetic cause of FSGS generally do not recur following kidney transplantation as the cause no longer exists. Therefore, if a patient with genetic FSGS develops FSGS post-transplantation, other causes (e.g., CNI toxicity) are implicated.

The CNI-FSGS group demonstrated high percentages of global sclerosis and IF/TA, caused by ischemia, and prominent endothelial cell injury was observed on EM. The glomerular and peritubular capillary endothelial cells are important targets for CNI, and circulating endothelial cells have been reported to be markers of endothelial injury due to CNI toxicity [5]. Conversely, the COL variant was also found in approximately 7% of cases in our study. A possible mechanism for epithelial cell injury in CNI toxicity is podocyte hypoxia due to severe hyaline deposition increasing hypoxia-inducible factor-1α (HIF-1α) with a subsequent increase in VEGF in the podocytes, leading to epithelial hyperplasia [23] (Fig. 4). However, epithelial hyperplasia does not occur in all cases of severe arteriolar hyalinosis, suggesting factors other than ischemia may be involved. Recent studies have demonstrated that accumulated oxidative stress in endothelial cells via the endothelin-1 (ET-1) signaling pathway may be involved in podocyte injury. CNI increases ET-1 in endothelial cells [24], and ET-1 exerts its effects via endothelin receptor A (ETRA) [24] which is localized primarily in renal vasculature smooth muscle cells under normal conditions. However, Daehn et al. reported that ET-1 derived from podocytes activates endothelial cells’ ETRA, causing the accumulation of oxidative stress in endothelial cell mitochondria, which in turn induces podocyte apoptosis (Fig. 4) [25, 26]. An increase in ETRA and oxidative stress in glomerular endothelial cells was also observed in cases of human FSGS [27].

The ABMR-FSGS group showed the highest incidence of the CEL variant among the groups, which had prominent endothelial injury. Endothelial injury in ABMR-related FSGS lesions may be due to the binding of DSA to donor endothelial cell membrane antigen (Fig. 4). DSA can cause indirect injury via complement fixation from classical pathway activation [28], where C4d is the final degradation product of C4 activation and remains stable on the endothelial cell membrane [29]. Conversely, in cases of ABMR with DSA without C4d deposition, DSA can cause direct capillary endothelium injury, or indirect injury via recruitment of inflammatory cells with Fc receptors [28].

In the UE-FSGS group, 72.6% of the cases were of the NOS variant. However, despite the mildest atherosclerosis among the groups, the percentage of IF/TA was the second highest in the CNI-FSGS group, suggesting that nonischemic mechanisms may also be involved. Glomerulonephritis was present in 27.5% of all cases, which was most common in the UE-FSGS group (38.9%). When other types of glomerulonephritis are present, segmental lesions are more easily regarded as their postinflammatory scars. In renal allografts, however, determining the origin of segmental lesions is often difficult because of factors, such as hyperfiltration from a single kidney, drugs, and rejection. Furthermore, the UE-FSGS group included many TCMR or reflux nephropathy cases with prominent IF/TA. Tubular cells secrete growth factors which activate fibroblasts producing an extracellular matrix [30], which causes tubulointerstitial fibrosis and secondary FSGS (Fig. 4). Moreover, a recent report showed that preexisting tubulointerstitial injury makes the glomerulus more susceptible to subsequent podocyte damage (Fig. 4) [31].

We examined semiquantitative urine protein by according to the histological variant in each group (Supplemental Fig. 2). Although it is difficult to draw conclusions, each group exhibited a similar or different trend compared to that reported in native kidneys [13]. We have previously reported that quantitative measurement of several parameters on electron micrographs was helpful in determining the degree of injury in epithelial and endothelial cells in native FSGS cases [8]. While the association between the degree of injury and proteinuria can be evaluated, we were not able to evaluate 24-h proteinuria in the present study. Unlike that performed in native kidney cases, 24-h urine collected to evaluate proteinuria was not performed in many of the renal transplant cases used in the study.

In conclusion, categorizing FSGS lesions in renal allografts by etiology revealed distinct clinicopathologic features, whereas it is difficult to identify the etiology from histological variants. This is because even in the same FSGS variants, there were differences in the degree of epithelial and endothelial injury, which may reflect different segmental lesion mechanisms. For example, the COL variant may occur by podocyte injury and also by endothelial injury or disruption of epithelium and endothelium crosstalk, which may relate to the molecular mechanisms involved, treatment and prognosis. Precise observation of FSGS lesions and understanding the pathogenesis of the segmental lesions may help in the diagnosis and clinical management of FSGS during renal transplantation.

### Supplementary Information

Below is the link to the electronic supplementary material.

Supplementary file1 (DOCX 2026 KB)

## Data Availability

All data analyzed during this study are included in this article and its supplementary information files. The datasets generated during the current study are not publicly available due to patients' privacy but are available from the corresponding author on reasonable request.
