# Supplementary material for: Clinicopathological differences in focal segmental glomerulosclerosis depending on the accompanying pathophysiological conditions in renal allografts
Source: Virchows Arch. 2023 Nov 18;483(6):809–19. doi: 10.1007/s00428-023-03703-6 (PMC10700464; doi:10.1007/s00428-023-03703-6)
Supplement: Supplementary file 1 — Supplementary file1 (DOCX 2026 KB) [file 428_2023_3703_MOESM1_ESM.docx]

**Supplemental Figure 1**

**The relationship of post-transplant duration, histological variant, and prognosis for cases with severe proteinuria in the recurrent-FSGS group**

When restricted to cases with severe proteinuria (UP ≥ 3), the proportion of the COL variant is approximately 70%. All cases within 3 months of transplantation were of the COL variant. The CEL variant was observed 4 months after transplantation and the NOS variant 7 months after transplantation. Two years after transplantation, the NOS variant became more common. The reintroduction of hemodialysis was more common in COL variant cases than in other variants.

FSGS, focal segmental glomerulosclerosis; COL, collapsing; CEL, cellular; NOS, not otherwise specified.

**Supplemental Figure 2**

**Scatter dot plots with medians (wide lines) and interquartile ranges (narrow lines) showing semiquantitative urinary protein levels determined with dipstick method** **by according to the histological variant in each group.**

**Supplemental Figure 3**

**Light (a) and electron micrographs (b,c) of MCD-like glomeruli obtained 5 weeks before the diagnosis of recurrent-FSGS, in the case presented in Fig. 2a and b.** Samples were obtained 20 days after transplantation. Urine protein was 27g/day. (a) No apparent change was detected in the glomeruli, whereas podocyte swelling (b) with foot process effacement was observed (c). (scale bars: a, 50 μm; b,c, 5 μm)

MCD, minimal change disease

Supplemental information

Supplemental Figure 1


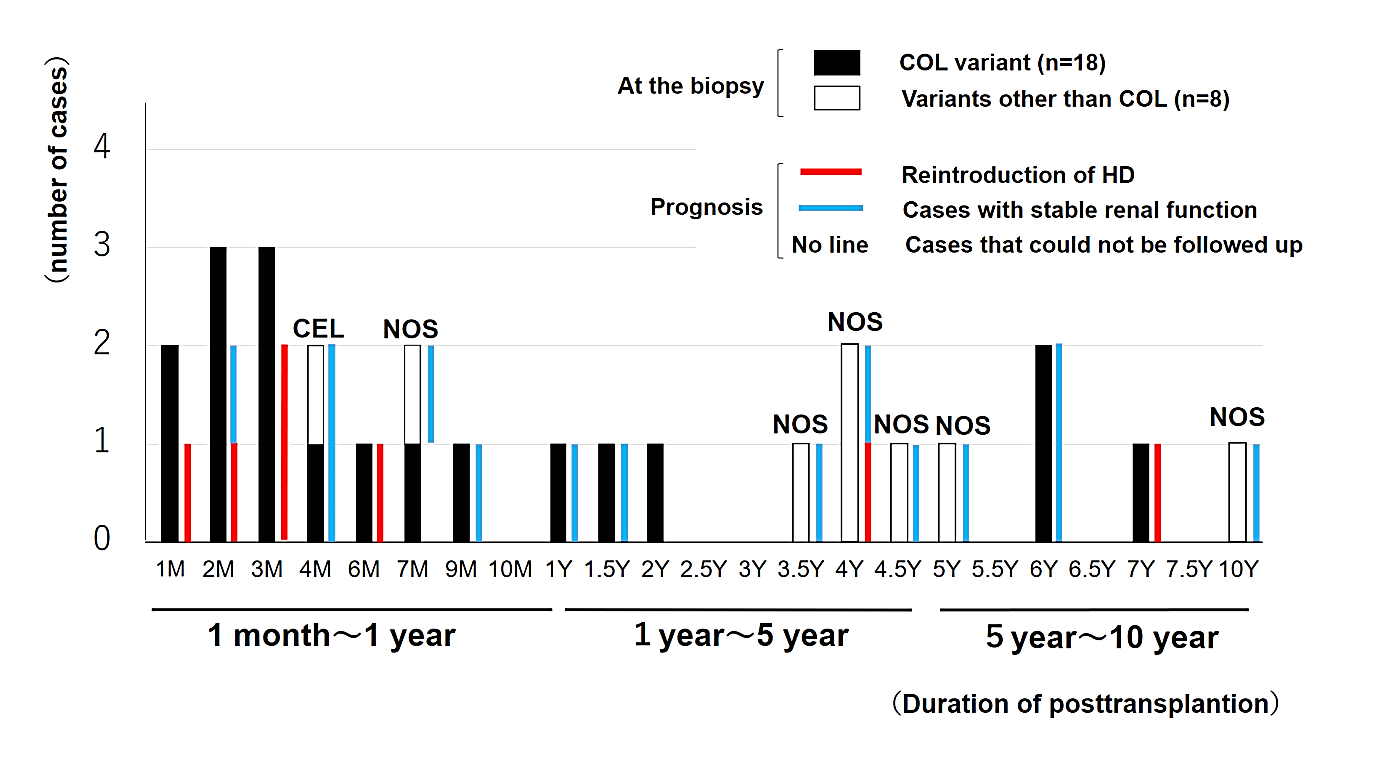


Supplemental Figure 2


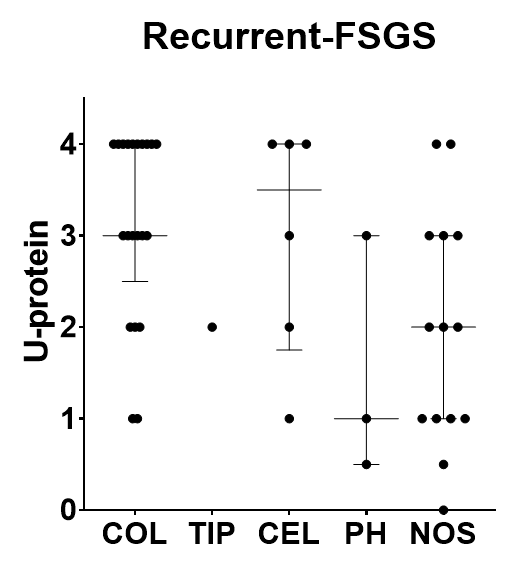
　
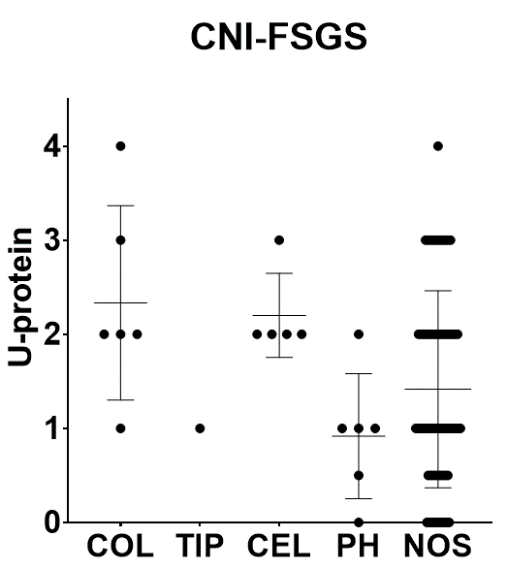


**(a)**

**(b)**


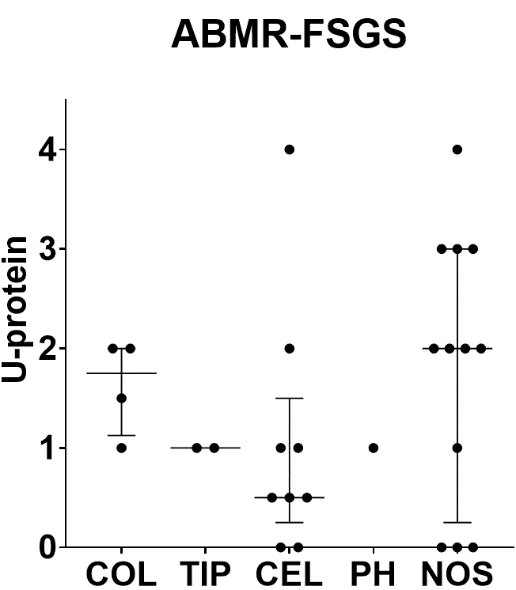
　
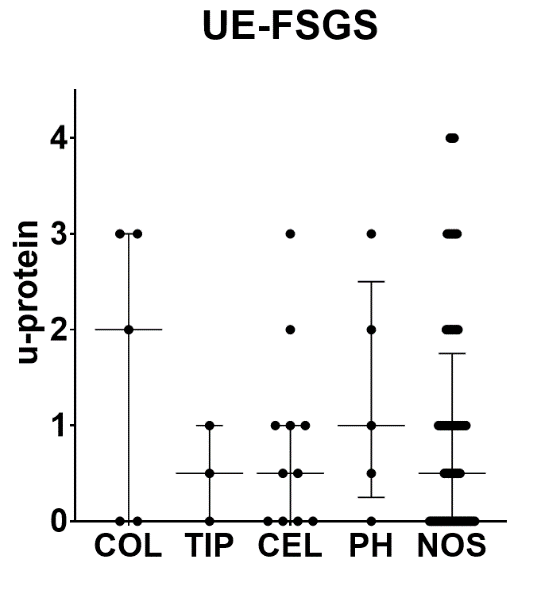


**(d)**

**(c)**

**Supplemental Figure 3**


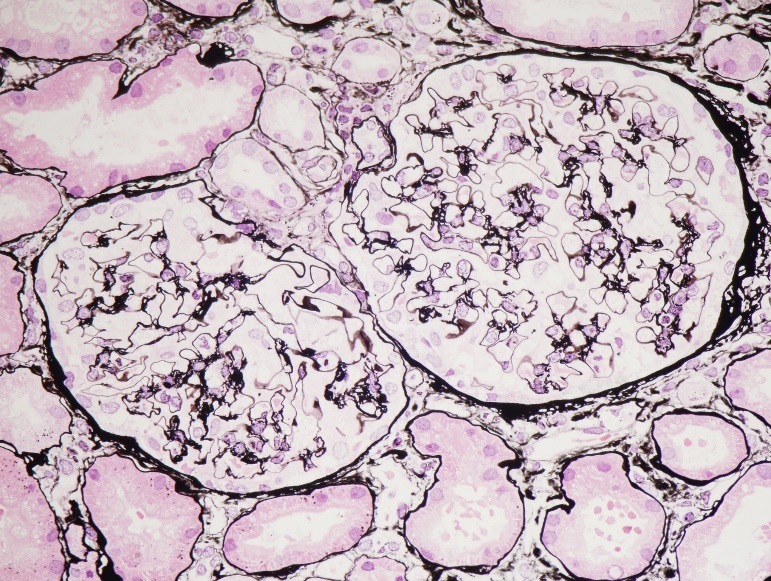

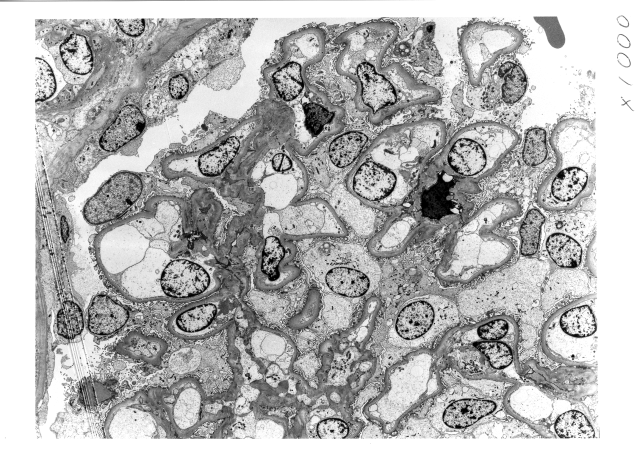


**b**

**a**


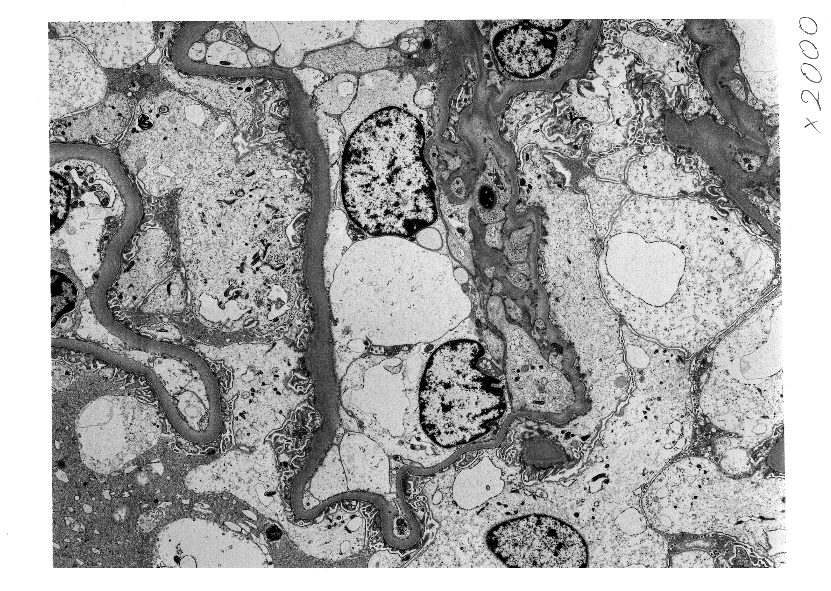


**c**
